# Lifestyle Risk Factors and Cardiovascular Disease in Cubans and Cuban Americans

**DOI:** 10.1155/2012/470705

**Published:** 2011-11-23

**Authors:** Melissa S. Burroughs Peña, Dhaval Patel, Delfin Rodríguez Leyva, Bobby V. Khan, Laurence Sperling

**Affiliations:** ^1^Division of Cardiology, Duke University Medical Center, Durham, NC 27710, USA; ^2^Division of Cardiology, Emory University, Atlanta, GA 30322, USA; ^3^Division of Cardiology, University Hospital V. I. Lenin, Holguin, Cuba; ^4^Atlanta Vascular Research Foundation, Atlanta, GA 30084, USA; ^5^Division of Cardiology, Emory University, Atlanta, GA, USA

## Abstract

Cardiovascular disease is the leading cause of mortality in Cuba. Lifestyle risk factors for coronary heart disease (CHD) in Cubans have not been compared to risk factors in Cuban Americans. Articles spanning the last 20 years were reviewed. The data on Cuban Americans are largely based on the Hispanic Health and Nutrition Examination Survey (HHANES), 1982–1984, while more recent data on epidemiological trends in Cuba are available. The prevalence of obesity and type 2 diabetes mellitus remains greater in Cuban Americans than in Cubans. However, dietary preferences, low physical activity, and tobacco use are contributing to the rising rates of obesity, type 2 diabetes mellitus, and CHD in Cuba, putting Cubans at increased cardiovascular risk. Comprehensive national strategies for cardiovascular prevention that address these modifiable lifestyle risk factors are necessary to address the increasing threat to public health in Cuba.

## 1. Introduction

According to the World Health Organization, cardiovascular disease (CVD) is the leading cause of death worldwide. Sixty percent of the global burden of coronary heart disease (CHD) occurs in developing countries [1]. While access to health care remains a barrier to prevention and treatment of CHD in many developing countries, Cuba is different. Cuba has had a system of socialized medicine and universal access to health care for over 40 years. In creating strategies to address the rising tide of CHD in Latin America and the Caribbean, it is useful to examine the Cuban experience to inform which approaches may be the most useful in resource-poor settings. While improving medical treatment for illnesses such as hypertension, dyslipidemia, and CHD will help to lower CHD mortality, prevention is a key component of turning the tide of the rising epidemic. Comprehensive CHD prevention includes examining the contribution of lifestyle risk factors to cardiovascular disease and creating strategies to modify diet, physical activity, and tobacco use in the population.

Beyond comparing Cuba to other developing countries, it is interesting to compare Cubans living in Cuba to those living in the United States. Several waves of immigration to the United States from Cuban have occurred since the Cuban Revolution in 1959. Although the majority of Cuban Americans tend to be white Cubans of higher socioeconomic background compared to the Cubans who remain in Cuba, comparing lifestyle factors such as diet, physical activity, and tobacco use provides an interesting perspective on the effect of immigration to the United States on cardiovascular disease risk. While it is assumed that acculturation to the American lifestyle conveys an increased risk for cardiovascular disease, one might posit that many Cubans have unhealthy lifestyle behaviors that are further amplified upon moving to the United States and improving their economic status. While the published data on CHD and CHD risk factors in Cubans and Cuban Americans are limited, the available literature will be reviewed in this paper.

## 2. Methods

Original articles, review articles, and reports that examine CHD and CHD risk factors in Cubans and Cuban Americans were searched and reviewed. Using PubMed, we searched the following terms: Cuba, Cubans, coronary heart disease, cardiovascular disease, hypertension, diabetes, tobacco, metabolic syndrome, obesity, diet, and physical inactivity. The bibliography of review articles were also searched for sources not found in PubMed. References suggested by colleagues in Cuba were also included in this paper. These references include articles published in Cuban journals and reports from the Cuban Ministry of Public Health which were found on their website (www.infomed.sld.cu). A total of 38 references were retrieved and included in this paper.

## 3. Diet

The fall of the Soviet Union in 1989 precipitated an economic crisis in the 1990's known as the Special Period. During the Special Period, patterns of nutrition and physical activity changed as food and energy resources became scarce. Vitamin and mineral deficiencies became more common and contributed to increased rates of anemia and optic neuropathy [2–4]. According to surveys conducted in Havana and Cienfuegos from 1980 to 2005, caloric intake decreased from 2899 kcal in 1988 to 1863 kcal in 1993. This change coincided with a rapid decline in type 2 diabetes mellitus and CHD mortality in the time period between 1997 and 2002, although morbidity was not impacted and continued to rise during this period [2, 3, 5].

Since the modest economic recovery, the increasing availability of inexpensive fast-food vendors in addition to the inconsistent accessibility and affordability of fresh fruits and vegetables is affecting the quality of the Cuban diet. However, in addition to food availability, food preferences should also be considered. In a recent survey of Cuban food preferences, red meat, ham, white bread, soft drinks, and processed food were identified as preferred food items. Ham and red meat were considered healthy by 85% and 90% of participants. This survey also reproduced earlier findings that the fruit and vegetable intake of most Cubans was inadequate [6, 7]. While cost was identified as a major factor in dietary choices, this survey demonstrates that if the barrier of cost was removed, food preferences would remain a barrier to healthy eating in Cuba. 

Data on Cuban American dietary preferences are less available. While there are no recent surveys of Cuban American dietary patterns, the 1982–84 HHANES survey describes dietary trends at that time. Seventy-three percent of Cuban American men and 75.7% of Cuban American women reported high “junk food” consumption [8]. While these data are based on self-reporting, they suggest that food preferences in Cuban Americans may not be that different than in Cubans.

## 4. Physical Inactivity

Physical inactivity is associated with a 19% increased risk of CHD when compared to individuals who regularly engage in vigorous physical exercise [9]. Physical inactivity in the elderly has been the subject of several studies in Havana. In a 2008 study of elderly patients in a polyclinic in Havana, physical inactivity was the most prevalent risk factor present in 74% of the sample [10]. Eighty-two percent of elderly women in another Havana study in 2011 reported low physical activity, a much greater percentage when compared to elderly women in Bridgetown, Barbados [11]. Another study of 1,905 elderly people in Havana found that decreased physical activity was a significant risk factor for overweight [12]. Some sources have estimated that presently 53–69% of all Cubans are sedentary [13]. Not only are adults inactive, but children are also becoming increasingly inactive with television and video games cited as the primary causes [14]. In a 2002 study of preschool children, only boys in rural areas and small towns met the minimum recommended daily physical activity as recommended by the Center for Disease Control (CDC) [15]. 

Physical inactivity is also common among Cuban Americans. The Miami Community Health Study examined CAD risk factors in African Americans, Cuban Americans, and white Americans living in Dade County, Florida, from 1991–1995. Self-reported physical activity varied according to sex. Forty-seven percent of Cuban American men reported physical activity less than 1000 kcal a week, which was greater than that reported in African American and White American men. However, Cuban American women reported less physical inactivity than African American and white American women with 51.6% undergoing less than 1000 kcal of physical activity per week [16].

## 5. Obesity

The global trends of poor dietary habits and low physical activity have resulted in the rising prevalence of obesity worldwide. Similar to the rest of the world, obesity has become a public health concern in Cuba. A 2001 national survey of a representative sample found that 29.7% of men and 31.5% of women were overweight and 7.9% of men and 15.4% of women were obese [17]. Other studies have suggested that 24.6% of women and 14.9% of men are obese [14]. These data suggest that obesity more greatly affects Cuban women than Cuban men. In examining obesity in rural Cuba, a 2008 study in the Isla de la Juventud province found that 31.3% of the adult population was estimated to be overweight and 13.4% was obese [18]. These data are similar to other studies done in rural Cuba, suggesting that while obesity is less prevalent in rural Cuba, it remains a public health concern throughout the island. 

Obesity in elderly Cubans has been compared to that in elderly populations in Latin America and the Caribbean. A 2003 study of the elderly population in the rural Cuban province of Pinar del Rio Cubans showed that they had lower body mass index (BMI), lower measurement of skin folds, lower waist circumference, and higher physical activity levels compared to elderly people in rural Chile and Mexico [19]. The average waist circumference in this small sample of elderly Cuban men was 81.3 cm and 80.5 cm in elderly Cuban women. This contrasts greatly to rural elderly Mexican men and women who had an average waist circumference 88.3 cm and 93.1 cm. In 2000, another study examined obesity in a larger sample of elderly Cubans in Havana and elderly Barbadians in Bridgetown [11]. The elderly Cubans had lower prevalence of obesity compared to elderly Barbadians affecting 12.7% of men and 30.7% of women. This comparison suggests that although obesity remains a public health concern throughout Cuba, urban and rural elderly Cubans are less obese than their counterparts in Latin America and the Caribbean. 

Obesity is increasingly affecting young adults and children. Cuba was among the four Latin American countries with the highest rates of obesity among 20–29-year olds in 1994, which of note was during the most difficult years of the Special Period [20]. In examining contemporary childhood obesity, the Comprehensive Childhood Study of 2004-2005 found that 10.2% of children are overweight and 8.8% are obese [14]. In 2005, the prevalence of excess weight in children <19 years of age in Havana was similar to that of 1972. However, there was a significant increase in high adiposity in children, many of whom had a normal BMI [21]. 

In the 1982–84 HHANES survey, nearly 30% of Cuban American men and 34% of Cuban American women were classified as overweight [22]. These data are similar to those seen in Mexican American men and women (30% and 39% resp.) and Puerto Rican American men and women (25% and 37%) [22]. Nine percent of Cuban American men and 15% of Cuban American women were obese, also comparable to other Latinos [23]. Overweight prevalence was higher among hypertensive Cuban Americans [24]. Of note, increased BMI in Cuban Americans was not associated with income, education, acculturation, or socioeconomic status [25]. However, this may be a limitation of the population sample in the HHANES study which underrepresented the poor. There are no available recent data on obesity in Cuban Americans.

## 6. Tobacco

Similar to other countries, tobacco use in Cuba is greater among men than among women. It is estimated that 40% of all men smoke tobacco every day, with a higher rate of 60% in middle age men. Moreover, 25% of women and 32% of men age greater than 60 smoke tobacco, a figure much greater than other Caribbean and Latin American countries [7]. Elderly Cubans smoke at higher rates than the rest of the population. A large study in Havana from 2011 estimated that 46.5% of elderly men and 21.5% of elderly women smoked tobacco [11]. Moreover, in the same study, an additional 31% of men and 15% of women were former smokers. The prevalence of tobacco use in Havana in this study was much greater than in Bridgetown, Barbados. In a 2003 case control study of patients with acute MI in Cuba, smoking was responsible for a third of the burden of acute myocardial infarction in Cuba [26]. 

In the HHANES 1982–1984 survey, smoking was reported in 23.9% of Cuban American women and 45.1% of Cuban American men [8, 24]. Within this cohort, 27.3% of Cuban American men were heavy smokers (greater than 20 cigarettes a day) compared to 11.9% of Cuban American women [8]. Birth cohort analysis of HHANES (1982–1984) data revealed that smoking rates among successive birth-cohorts increased substantially in Cuban American women [27]. Subsequent studies from the early 1990's reported a lower prevalence of tobacco use. Smoking rates among men ranged from 21.4 to 30.3% and in women 11.6–25.9% [16, 28, 29]. Among smokers, Cuban American men had the highest mean daily cigarette use (17.8 cigarettes a day) compared to other Latino ethnic groups [29].

## 7. Type 2 Diabetes and the Metabolic Syndrome

With poor dietary habits increasing and physical activity declining, more Cubans are at risk for developing type 2 diabetes mellitus and the metabolic syndrome. The metabolic syndrome, which consists of hypertension, glucose intolerance, dyslipidemia, and abdominal obesity, is increasingly being recognized as an independent risk factor for CHD beyond the sum of the risk conveyed by its individual components [30]. There are few studies that have examined the prevalence of the metabolic syndrome in Cuba. A cross-sectional study in the city of Cienfuegos found the prevalence of the metabolic syndrome according to the NCEP ATP III definition in adults to be 22% (95% confidence interval 14.5–28.8) [31]. However, in adults greater than 40 years old, the prevalence of the metabolic syndrome is 44% [31]. The prevalence of the metabolic syndrome in rural areas of Cuba is unknown.

Moreover, there are little data on the epidemic of type 2 diabetes mellitus in Cuba. In one survey of individuals age 60 and older, 15% of participants reported having type 2 diabetes mellitus [7]. Another study of elderly individuals in Havana reported type 2 diabetes mellitus was more prevalent in women compared to men [11]. While 19.9% of elderly women had type 2 diabetes mellitus, only 7.3% of men were diabetic. Other studies have estimated a national prevalence of type 2 diabetes ranging from 4.6 to 17% [6, 13, 31]. 

A few small observational studies have been done in order to gain insight into the impact of the metabolic syndrome on the health of Cubans. A 2005 observational study of patients in a Havana hospital examined the comorbidities of patients that died in the intensive care unit. Of the 149 patients that died during the study, 88 patients (32.9%) had the metabolic syndrome as defined by NCEP ATP III. The metabolic syndrome was a comorbidity in all deaths attributed to cardiogenic shock, myocardial infarction, and mesenteric thrombosis and was present in greater than 60% of individuals that died from ischemic and hemorrhagic stroke [32].

Data from the 1982–84 HHANES reveal that 16% of Cuban Americans aged 45–74 had type 2 diabetes mellitus, which was slightly greater than non-Latino Whites (12%), but significantly less than Mexican Americans (24%) and Puerto Rican Americans (26%) [33]. From 1996 to 1997, diabetes-related mortality was twice as high for Mexican and Puerto Rican Americans than Cuban Americans in persons aged greater than 35 [34].

When combining HHANES data with the National Health and Nutrition Examination Survey (NHANES), age-standardized prevalence of diabetes in Cuban Americans is only 9.3% [35]. 

A recent observational study demonstrated that among 161 nondiabetic Cuban Americans in South Florida, 41% met criteria for the metabolic syndrome as defined by NCEP ATP III [36]. Moreover, Cuban Americans with the metabolic syndrome had elevated high-sensitivity CRP levels that may also indicated an increased risk for type 2 diabetes mellitus and cardiovascular diseases [36]. This observation has yet to be further explored.

## 8. Coronary Heart Disease

The increasing prevalence of obesity, type 2 diabetes mellitus, and the metabolic syndrome is putting more Cubans at risk for CHD. Obesity is not only associated with an increased risk of hypertension, type 2 diabetes mellitus, and hyperlipidemia, but also earlier age at which first non-ST elevation myocardial infarction occurs [37]. Twenty-six percent of all deaths in 2002 were attributed to heart disease [7]. In a 2001 study conducted in a polyclinic in Havana, CHD was one of the most prevalent chronic diseases in patients age 60 and older, third to hypertension and type 2 diabetes mellitus [10]. Of all of the heart transplants done in Cuba from 1985 to 2005, 57.5% of the cases were due to CHD [38]. While the prevalence of CHD has continued to rise over the last four decades, mortality related to ischemic heart disease has been declining since the 1990's [39]. From 1991 to 2000, the percentage of deaths from ischemic heart disease secondary to MI decreased from 73% to 54% [39]. Public health initiatives and advances in care for patients with acute myocardial infarction have contributed to this decline. However, when Cuba was compared to 9 other Latin American countries, the United States, and Canada in 2000, Cuban women had the highest CHD mortality rates and Cuban men ranked third behind that of Venezuela and the United States [7].

Similar to the distribution of CHD risk factors, CHD affects urban areas more than rural areas. The rate of cardiovascular disease is 40% higher in urban areas when compared to rural areas [7]. In the year 2008, the city of Havana had the largest cardiovascular-related mortality at 256.6 deaths per 100,000 compared to the Isla de la Juventud whose cardiovascular-related mortality was the lowest at 105.0 per 100,000 [40]. 

Like many Latino ethnic groups, there is very little data on the prevalence and incidence of CHD in Cuban Americans. Data from the National Health Interview Survey- Multiple Causes of Death (NHIS-MCD), 1986–1995, reveal that cardiovascular disease is the leading cause of death for Cuban Americans [41]. Rates of cardiovascular-related mortality are similar to that of white Americans but less than that of Mexican Americans, Central Americans, and South Americans [41].

## 9. Conclusion

The Cuban dietary and physical activity patterns convey an increased risk for CHD even before immigrating to the United States. While pharmacological treatment of CHD and CHD risk factors such as hypertension and dyslipidemia is essential in confronting this epidemic, Cuba has already achieved much success in this area. In a 2008 study conducted in Cienfuegos, 40% of the participants with hypertension were at or below goal blood pressure of 140/80 mmHg, which was greater than national rates in the United States (34%) [42, 43]. Yet despite achieving rates of hypertension control greater than that of developed countries, Cuba has to confront to challenge of modifying the lifestyle of its population in order to provide more comprehensive cardiovascular prevention. This includes creating more programs and space for physical activity and recreation in addition to making healthy foods like fresh fruits and vegetables affordable and available. These changes extend much further than the Ministry of Public Health, but also include education, urban planning, and agriculture. Cuba is not alone in facing this challenge, but as Cuba creates solutions in the setting of limited resources, it can serve as a model for the rest of the developing world.
